# Role of ACTH and Other Hormones in the Regulation of Aldosterone Production in Primary Aldosteronism

**DOI:** 10.3389/fendo.2016.00072

**Published:** 2016-06-27

**Authors:** Nada El Ghorayeb, Isabelle Bourdeau, André Lacroix

**Affiliations:** ^1^Department of Medicine, Division of Endocrinology, Centre de Recherche du Centre hospitalier de l’Université de Montréal (CRCHUM), Université de Montréal, Montréal, QC, Canada

**Keywords:** ACTH, aldosterone regulation, melanocortin type 2 receptor, aberrant G-protein coupled receptors, primary aldosteronism

## Abstract

The major physiological regulators of aldosterone production from the adrenal zona glomerulosa are potassium and angiotensin II; other acute regulators include adrenocorticotropic hormone (ACTH) and serotonin. Their interactions with G-protein coupled hormone receptors activate cAMP/PKA pathway thereby regulating intracellular calcium flux and CYP11B2 transcription, which is the specific steroidogenic enzyme of aldosterone synthesis. In primary aldosteronism (PA), the increased production of aldosterone and resultant relative hypervolemia inhibits the renin and angiotensin system; aldosterone secretion is mostly independent from the suppressed renin–angiotensin system, but is not autonomous, as it is regulated by a diversity of other ligands of various eutopic or ectopic receptors, in addition to activation of calcium flux resulting from mutations of various ion channels. Among the abnormalities in various hormone receptors, an overexpression of the melanocortin type 2 receptor (MC2R) could be responsible for aldosterone hypersecretion in aldosteronomas. An exaggerated increase in plasma aldosterone concentration (PAC) is found in patients with PA secondary either to unilateral aldosteronomas or bilateral adrenal hyperplasia (BAH) following acute ACTH administration compared to normal individuals. A diurnal increase in PAC in early morning and its suppression by dexamethasone confirms the increased role of endogenous ACTH as an important aldosterone secretagogue in PA. Screening using a combination of dexamethasone and fludrocortisone test reveals a higher prevalence of PA in hypertensive populations compared to the aldosterone to renin ratio. The variable level of MC2R overexpression in each aldosteronomas or in the adjacent zona glomerulosa hyperplasia may explain the inconsistent results of adrenal vein sampling between basal levels and post ACTH administration in the determination of source of aldosterone excess. In the rare cases of glucocorticoid remediable aldosteronism, a chimeric CYP11B2 becomes regulated by ACTH activating its chimeric CYP11B1 promoter of aldosterone synthase in bilateral adrenal fasciculate-like hyperplasia. This review will focus on the role of ACTH on excess aldosterone secretion in PA with particular focus on the aberrant expression of MC2R in comparison with other aberrant ligands and their GPCRs in this frequent pathology.

## Introduction

Primary aldosteronism (PA) was first described in patients with unilateral aldosterone-producing adenomas (1). It is characterized by increased aldosterone secretion causing salt retention, increased urinary potassium excretion, relative hypervolemia, suppressed plasma renin activity (PRA), and hypertension. PA is the most common curable form of secondary hypertension as it affects 4.3% of the general hypertensive population, 9.5% of patients referred to hypertension clinics (2), and up to 20% of those with resistant hypertension (3). PA is most often secondary to bilateral adrenal hyperplasia (BAH; 50–70% of cases) or to an aldosterone-producing adenoma (APA; 30–50% of cases) (4). The classical concept that a unique unilateral aldosteronoma is the causative lesion responsible for a high proportion of this surgically curable form of PA was recently challenged by the identification of zona glomerulosa (ZG) hyperplasia and nodulation adjacent to aldosteronomas when resected adrenals are examined carefully (5–7).

In order to prevent cardiovascular, metabolic and renal morbidities, early diagnosis and management of PA are mandatory (8–10). Unilateral adrenalectomy (UA) provides superior benefit compared to medical therapy in lateralized PA in terms of cardiovascular outcomes (11, 12), quality of life (12), and all-cause mortality (13); however, in BAH, pharmacological blockade of aldosterone excess using mineralocorticoid receptor antagonists, such as spironolactone or eplerenone, is the recommended treatment (4). Therefore, subtyping of PA is required to direct patients to surgical vs. medical therapy (4). To date, adrenal vein sampling (AVS) is the gold standard to differentiate lateralized from bilateral sources of PA (4) because adrenal imaging provides poor specificity in detecting lateralized cases (14) except in patients <35 years old (15).

In PA excess, plasma aldosterone concentration (PAC), despite suppressed renin activity, is not really autonomous, as frequently stated. It could be autonomous if it was solely or constitutively regulated by somatic and germline mutations of various ion channels genes regulating intracellular ionic homeostasis and cell membrane potential as reviewed elsewhere (16). In fact, several autocrine/paracrine hormones and regulatory mechanisms (17) activate variable levels of aberrant eutopic or ectopic receptors (18), which regulate aldosterone secretion either in unilateral adenomas or in BAH. In this review, we will focus on the role of one of these hormones, the adrenocorticotropic hormone (ACTH), in stimulating aldosterone secretion in normal and pathologic conditions and briefly mention others which play similar roles in PA.

## Normal Physiology of the Renin–Angiotensin System

Renin is an enzyme produced primarily by the juxtaglomerular apparatus of the kidney and its release is the rate-limiting step in the regulation of the RAS (19, 20); it is controlled by four factors: (1) the macula densa comprises chemoreceptors for monitoring the sodium and chloride loads present in the distal tubule, (2) juxtaglomerular cells acting as pressure transducers that sense stretch of the afferent arteriolar wall and thus renal perfusion pressure, (3) the sympathetic nervous system (SNS), which increases the release of renin, particularly in response to upright posture, in addition to (4) inhibiting factors, including K^+^, Ca^++^, angiotensin II, and atrial natriuretic peptides (19).

**Figure 1:**
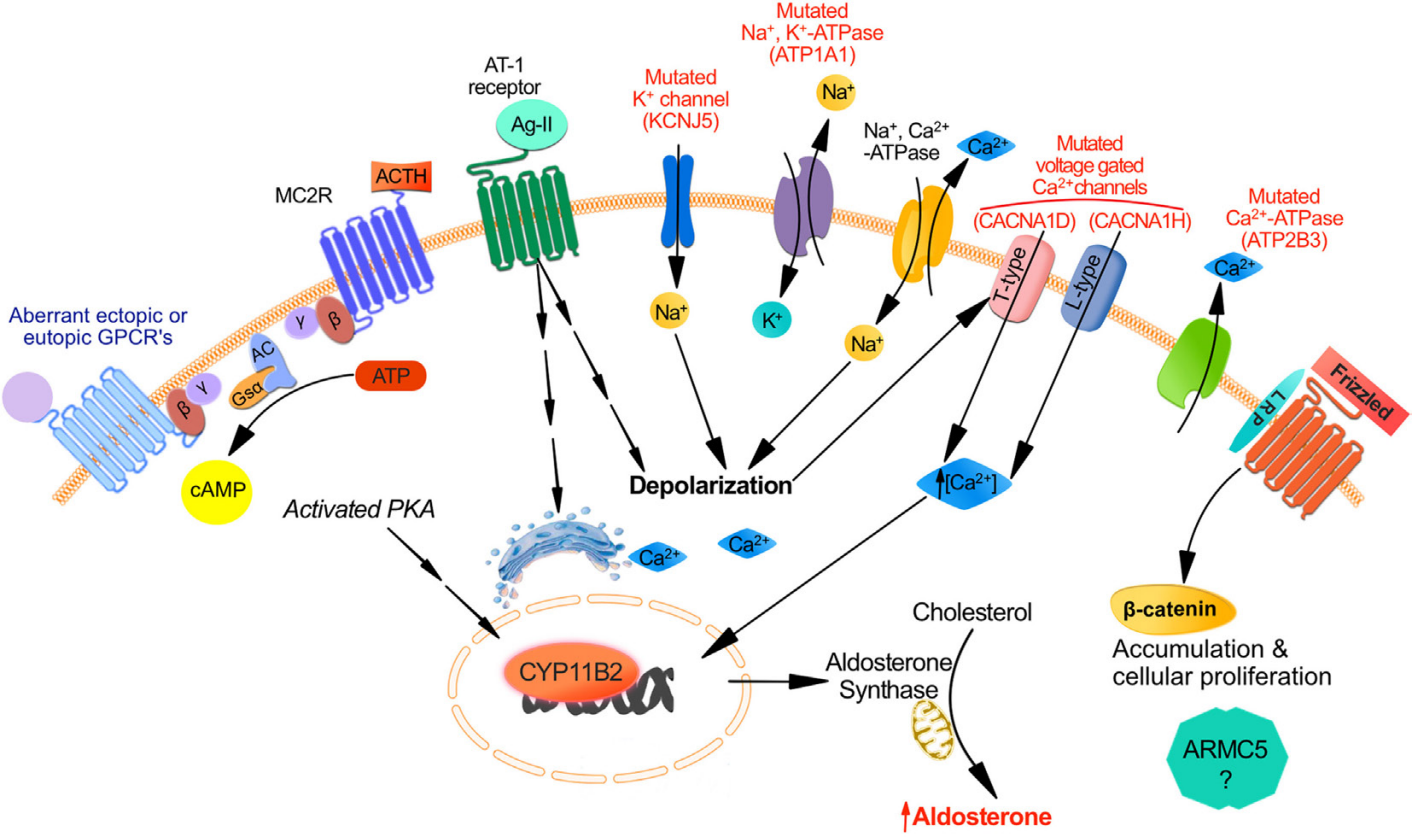
**Mechanisms responsible for aldosterone synthesis in zona glomerulosa cells under normal physiological conditions and excess production in primary aldosteronism**. The strongly negative resting membrane potential of zona glomerulosa (ZG) cells under resting physiological conditions is maintained by the concentration gradient of K^+^ between the intracellular and extracellular space, which is generated by the activity of the Na^+^, K^+^-ATPase. Angiotensin II and increased K^+^ lead to cell membrane depolarization, which opens voltage-dependent Ca^2+^ channels. Furthermore, Angiostensin II acts through the Angiotensin II type 1 receptor (AT1R) inducing Ca^2+^ release from the endoplasmic reticulum. Consequently, the increase in intracellular Ca^2+^ concentration activates the calcium signaling pathway, which triggers activation of CYP11B2 transcription. The role for ACTH in the regulation of aldosterone secretion whether in normal physiology or in PA is in part determined by the level of expression of ACTH receptors (MC2R) in ZG cells. MC2R which is a GPCR coupled to the stimulatory Gsα subunit may induce an increase of intracellular cAMP concentration which activates protein kinase A thereby increasing CREB phosphorylation and CYP11B2 transcription. Aberrant expression of other GPCR may also be responsible for aldosterone excess despite a suppressed renin angiotensin system: eutopic GPCR include those for serotonin (5-HT_4_R); ectopic GPCR include those for glucose-dependent insulinotropic peptide (GIPR), luteinizing hormone/human chorionic gonadotropin (LH–hCG R), β-adrenergic receptors (β-AR), vasopressin (V1-AVPR) glucagon (glucagon receptor), TRH (TRH R), and Endothelin-1 ET_A_ and ET_B_ receptors. Other mechanisms implicated in PA involve somatic and germline mutations in ion channels genes regulating intracellular ionic homeostasis and cell membrane potentials: increase intracellular Na^+^ concentrations and cell membrane depolarization result from *KCNJ5* gain-of-function mutations affecting GIRK4 and *ATP1A1* mutations of the Na^+^, K^+^-ATPase. Direct increase of intracellular Ca^2+^ concentrations could also result from mutations in *ATP2B3* encoding for the plasma membrane Ca^2+^-ATPase, mutations in *CACNA1D* affecting the Cav1.3 subunit of the L-type voltage-gated calcium channel or *CACNA1H* affecting the Cav3.2 subunit of the voltage-gated calcium channel. Finally dysregulation in cellular proliferation/apoptosis accelerating adenoma formation could be due either to activation of the Wnt/β-catenin pathway or to gene mutations such as *ARMC5* although the mechanism of the latter mutation is not fully elucidated.

The action of renin on angiotensinogen, synthesized in the liver, generates angiotensin I (19). Angiotensin-converting enzyme (ACE), localized in cell membranes particularly of the lung, cleaves angiotensin I into angiotensin II, which is the main biologically active angiotensin (19). Angiotensin II functions through the AT-1 receptor (AT1R) to maintain normal extracellular volume and blood pressure by increasing aldosterone secretion from the ZG *via* increased transcription of CYP11B2 (aldosterone synthase) (Figure 1) as well as constricting vascular smooth muscle, releasing norepinephrine, and epinephrine from the adrenal medulla, enhancing the activity of the SNS and finally promoting the release of vasopressin (19).

Zona glomerulosa cells are organized in rosette structures that spontaneously generate periodic depolarizing changes in membrane potential that are modulated in frequency by angiotensin II and extracellular K^+^ (21, 22); Angiotensin II induces cell membrane depolarization most probably due to a Gi-mediated shift in the voltage dependence of channel activation toward more negative potentials thereby increasing intracellular Ca^2+^ signal, which stimulates hormone-sensitive lipase and steroidogenic acute regulatory protein (StAR). Another mechanism by which angiotensin II binding to AT1R stimulates aldosterone secretion implicates activating the phospholipase C/inositol 1,4,5-trisphosphate pathway, releasing Ca^2+^ stores from the endoplasmic reticulum, and activation of T-type voltage-gated Ca^2+^ channels (23) (Figure 1).

Dopamine, atrial natriuretic peptide, and heparin inhibit aldosterone secretion. The secretion of aldosterone is restricted to the ZG because of zone-specific expression of aldosterone synthase (CYP11B2), which is regulated by the activation of calcium signaling (24).

## Pathophysiology of Primary Aldosteronism

The binding of free aldosterone to the mineralocorticoid receptor in the cytosol of epithelial cells (24), principally in the kidney, controls potassium homeostasis and maintains normal intravascular volume by increasing intestinal and renal Na^+^ and Cl^−^ absorption and reabsorption, respectively. Increased production of aldosterone in PA results in sodium retention, hypertension, and can also result in hypokalemia (20). In addition to the two most common subtypes of PA (BAH in 50–70% of the cases and APA in 30–50%), less frequent causes include primary (unilateral) adrenal hyperplasia (5%), aldosterone-producing adrenocortical carcinoma (<1%), familial hyperaldosteronism (1%), and ectopic aldosterone-producing adenoma or carcinoma (<0.1%). The mechanisms implicated in the pathophysiology of PA are not fully elucidated. Somatic and germline mutations in ion channels genes regulating intracellular ionic homeostasis and cell membrane potential were described in sporadic APA and type-III familial PA (25–28) (Figure 1). Somatic mutations in the potassium channel gene *KCNJ5* are found in almost 30–40% of aldosteronomas and alter channel selectivity allowing enhanced Na^+^ conductance. Na^+^ influx results in cell depolarization, the activation of voltage-gated Ca^2+^ channels, aldosterone production, and cell proliferation (25, 29). Somatic and germline mutations in *CACNA1D* gene encoding a voltage-gated calcium channel result in channel activation and less depolarized potentials causing increased Ca^2+^ influx, aldosterone production and cell proliferation in affected ZG cells (27, 30). Mutations in *ATP1A1* gene (encoding the Na^+^/K^+^ ATPase α subunit) and *ATP2B3* gene (encoding the plasma membrane Ca^2+^ ATPase) were identified in 5.2 and 1.6%, respectively of patients in a series of APA (26). Mutations in *CACNA1H* gene, which encodes a voltage-gated calcium channel (Cav3.2) were discovered in children with PA; they result in impaired channel inactivation and activation at more hyperpolarized potentials, producing increased intracellular Ca^2+^ and aldosterone excess (31). Different mutations in the genes described above are found in different aldosterone-producing nodules from the same adrenal, suggesting that somatic mutations are independent events (32, 33).

No mutations of any of the above ion channel genes were found in BAH or in ZG hyperplasia adjacent to the dominant aldosteronomas (26, 29, 32, 33); these findings suggest that nodule formation and excess aldosterone production are two dissociated events, implying a two-hit hypothesis for APA formation (16, 34). The first hit causing a unilateral aldosteronoma or a dominant nodule adjacent to ZG hyperplasia may result from a somatic mutation in one of the genes described above, at least in approximately 60% of cases. Possible causes of the second hit that results in dysregulation in cellular proliferation/apoptosis accelerating adenoma formation could be due either to activation of the Wnt/β-catenin pathway (35, 36), PKA pathway, or to gene mutations such as *ARMC5* (37) (Figure 1). However, the pathophysiology of progression from normal adrenal to APA and the causes of diffuse bilateral hyperplasias, either as BAH or in mild form adjacent to the dominant aldosteronoma, are still unknown. Aldosterone-producing cell clusters (APCCs), which have increased expression of CYP11B2, are nests of cells below the adrenal capsule. They protrude into cortisol-producing cells that are usually negative for CYP11B2 expression. Nishimoto et al. found that APCCs are common in normal adrenals, and they harbor a different mutational spectrum compared to APA suggesting that APCCs could be a precursor for APA (38). In addition, several hormones activating variable levels of eutopic or ectopic aberrant receptors (18) (Figure 1) as well as autocrine and paracrine regulatory mechanisms (17) can increase aldosterone secretion in PA (either APA or BAH) independently from the suppressed RAS (see later section).

## Role of ACTH in Aldosterone Production in Normal Physiology

Adrenocorticotropic hormone can stimulate aldosterone secretion acutely and transiently under normal conditions, but to a lesser extent than angiotensin II and potassium. ACTH is a 39-amino-acid peptide, which results from the cleavage of its proopiomelanocortin (POMC) precursor by prohormone convertases PC1/3 and may be further cleaved by PC2 to generate α-melanocyte-stimulating hormone (α-MSH) (39, 40). It is mainly produced in the anterior pituitary corticotropes, but is also produced in brain, adrenal medulla, skin, and placenta (41–43). ACTH can induce aldosterone production at lower doses than the ones needed for cortisol and DHEA production (44). Furthermore, ACTH stimulates aldosterone production acutely and sometimes chronically.

### Acute Effects of ACTH

The initial binding of ACTH to its specific melanocortin type 2 receptor (MC2R) stimulates both cortisol and aldosterone secretion. MC2R (45) is a seven transmembrane domain receptor that belongs to the family of melanocortin receptors (MCRs) (45). Five MCRs constitute a distinct family of G-protein coupled hormone receptors (GPCR); MC2R is the smallest MCR and GPCR (45, 46). MC2R is expressed in zona fasciculata (ZF) and ZG cells (47). The binding of ACTH to its MC2R induces the dissociation of Gs-α subunit and activation of adenylate cyclase (AC) that generates cAMP from ATP (48) (Figure 1). cAMP molecules bind to specific domains of the regulatory subunits of protein kinase A (PKA) thereby dissociating the tetramer and releasing the catalytic subunit (PRKACA) from its inactivating regulatory subunits. Activated PRKACA phosphorylates and activates steroidogenic acute regulatory protein (StAR) as well as cAMP response element binding protein (CREB), thereby increasing StAR expression. On the other hand, activation of the PKA pathway induces a slow but sustained calcium influx through the L-type calcium channels. The subsequent increase in intracellular calcium activates calcium/calmodulin-dependent protein kinase and steroidogenesis (49, 50).

### Chronic Effects of ACTH

In contrast to *in vivo* studies that suggest that ACTH is a short-term stimulator of aldosterone production, *in vitro* studies showed that ACTH can act as a major stimulus of aldosterone secretion. Continuous intravenous infusion of ACTH leads to a transient stimulation of aldosterone secretion, whereas its pulsatile administration leads to a sustained stimulation of aldosterone up to 72 h (51). Moreover, chronic exposure to ACTH (2 days or more) leads to transformation of ZG cells into ZF-like cells with elongated mitochondria with lamellar and tubular cristae becoming round with ovoid cristae; at the functional level, the synthesis of angiotensin II receptors, steroidogenic enzymes, and their products is altered (52–56).

## Role of ACTH in Excess Aldosterone Secretion in PA

### Diurnal Rhythmicity of Aldosterone

In recumbent normal subjects on a regular diet, the circadian rhythm of PAC is regulated by the activity of plasma renin independently of ACTH (57). In contrast, patients with PA have a circadian rhythm of PAC mediated by changes in ACTH rather than by the suppressed plasma renin–angiotensin II levels (58). Several groups described that PAC falls following overnight sleep when ACTH levels are low despite upright posture or angiotensin II infusion. Similarly, they noted a marked increase in PAC shortly after ACTH administration (59–63), which was higher compared to normal controls or patients with essential hypertension (64, 65). Furthermore, abolition of diurnal rhythm by dexamethasone in PA demonstrates the impact of ACTH on adrenal steroidogenesis (66). Administration of dexamethasone 0.75–2.0 mg per day for 2 days decreased aldosterone levels by a mean of 49% in a group of 15 patients with aldosteronomas; in 33%, the suppression was greater than 80% (67).

### ACTH Role in Familial Hyperaldosteronism

Familial hyperaldosteronism (FH) type-1, previously known as glucocorticoid-remediable aldosteronism (GRA), was first described as a form of hyperaldosteronism relieved by dexamethasone (68). It is suspected in young PA patients whose relatives suffer from cerebrovascular accidents. It is an autosomal dominant disease whereby the promoter of the chimeric 11β-hydroxylase/aldosterone synthase gene belongs to the 5′ end of CYP11 B1 (11β hydroxylase) and drives the expression of the 3′ end of CYP11 B2 (aldosterone synthase) ectopically in ZF cells under the main regulation by ACTH (69); in these patients, dexamethasone usually decreases aldosterone secretion by more than 80% or to <4 ng/dL (67), but the diagnosis is now performed using genetic analysis. In contrast to FH type-1, FH type-2 is defined as PA in a patient with a first-degree relative (parent/sibling/offspring) with established PA but without FH type-1 gene rearrangement. Linkage analysis has mapped FH type-2 to chromosome 7p22 but no responsible gene has been identified yet (70). The prevalence of FH type-2 in PA is higher (1.2–6%) than FH type-1 (≤1%). The FH type-3 and -4 are not regulated by ACTH stimulation, but they are caused by germline mutations in *KCNJ5* (71) and *CACNA1D/CACNA1H* (30, 31) genes, respectively.

### ACTH Suppression or Stimulation Tests Can Reveal the Presence of PA

Based on the rationale that ACTH plays a more important role in PA than in normal subjects or those with essential hypertension, investigators in Athens compared the classical saline infusion test (SIT) to postdexamethasone SIT in 151 patients with single adrenal adenomas and detected almost double rate of aldosterone hypersecretion following dexamethasone administration (24 vs. 12%) (72). Similarly, they used a combined fludrocortisone–dexamethasone suppression test (FDST), which is a modification of the classic confirmatory fludrocortisone suppression test (FST) for the diagnosis of PA; it involves the administration of dexamethasone to hypertensives patients at midnight of the last day of the FST in order to eliminate the stimulatory effect of ACTH on aldosterone secretion. They demonstrated that the prevalence of PA rises from 5 to 13% with the usual diagnostic tests to 28.7–31% when using FDST; mineralocorticoid receptor blockade resulted in significant improvement in blood pressure in these patients (73–75). The same group administered an ultralow-dose (0.03 μg) ACTH to 113 hypertensives without PA: the 30 patients (27%) who exhibited an aldosterone hyperresponse had significantly higher PAC, ARR, and PAC/ACTH ratio in the treadmill test; normalization of blood pressure by mineralocorticoid antagonists in these patients was also evident compared to the group of hypertensive not sensitive to ACTH/stress (76). Therefore, the benefit of mineralocorticoid blockade could extend even to hypertensive patients without confirmed PA who present an aldosterone hyperreseponse to ACTH/stress, this category of hypertenives harboring a mild form of BAH. In contrast, another group examining the diagnostic accuracy of ACTH test in 158 hypertensive patients found that it was not very effective in differentiating between APA patients and non-PA patients (77).

### Use of ACTH to Identify the Source of Aldosterone Excess

Many efforts were conducted to find an easier and cheaper test than adrenal vein sampling (AVS), which is available only in tertiary care center with experienced angioradiologists to distinguish between lateralized and bilateral sources of aldosterone. Differential increase in PAC during upright posture was suggested to be a valuable tool to distinguish APA from BAH, but further studies showed that several APA and BAH had similar rise in PAC to upright posture (78). APA whether responsive or not to angiotensin II was found to be more sensitive to ACTH stimulation resulting in larger increase of PAC than in patients with BAH or essential hypertension (79–81). BAH patients also displayed increased response of PAC to ACTH administration than normal subjects or patients with essential hypertension (82). PAC increased more after ACTH bolus in the APA group compared with BAH group, which had an intermediate increase compared to normal controls (18, 66). A study in which patients received dexamethasone (1 mg) the evening before receiving i.v. injection of 50 IU of ACTH showed that the exaggerated PAC response was higher after 120 min in patients with APA than in BAH (83). It was suggested that this could be used for identifying the etiology subtype; however, significant overlap was present between APA, unilateral hyperplasia, and BAH cases and using an optimal cutoff value of the aldosterone >78 ng/dL for APA, provided a sensitivity of 76.8% and a specificity of 87.2% (83).

Kline et al. studied 65 patients with confirmed PA who were divided by histology into confirmed lateralized and non-lateralized; PAC in inferior vena cava (IVC) sampled during AVS before and after cosyntropin infusion was analyzed. Baseline and peak IVC aldosterone was higher in lateralized patients (APA) but incremental aldosterone rise was much greater in subjects with bilateral hyperplasia (84). This shows that ACTH can regulate APA as well as BAH, but that the effects are more pronounced in APA.

### Role of ACTH Stimulation during Adrenal Venous Sampling

The usefulness of ACTH stimulation in the conduct of AVS procedure is controversial and remains a matter of debate because of conflicting results. Some centers use cosyntropin infusion or bolus in order to minimize stress-induced or spontaneous fluctuations in aldosterone secretion when performing sequential non-simultaneous AVS, to maximize the gradient of cortisol from the adrenal vein to the inferior vena cava, and to maximize aldosterone secretion from an APA (85). In contrast, other groups found that ACTH-stimulation of aldosterone production from the contralateral gland or adjacent hyperplasia may reduce the gradient of aldosterone production resulting in incorrect lateralization (86, 87). The effect of both continuous ACTH infusion and bolus on the performance and interpretation of AVS in confirmed PA patients was investigated (88). Both methods lead to a significant increase in selectivity index for the right adrenal vein and ACTH bolus for the left adrenal vein. Lateralization index was not significantly affected after continuous ACTH infusion and i.v. bolus. In 88 and 78% of the patients, the diagnosis obtained was the same before and after ACTH infusion and i.v. bolus, respectively (88). Recently, our group demonstrated that ACTH increased selectivity on both sides from 66.7% in basal samples to 91.8% poststimulation. A discordance of lateralization between basal and post-ACTH values was observed in 28% of cases, mostly lateralized cases basally that became bilateral post ACTH (87). The variation in the response to ACTH stimulation could be due to the variable expression of MC2R in APA (see later) (18). Careful examination of the levels of aldosterone in the adrenal vein contralateral to the dominant or lateralized APA and pathology confirmed the frequent presence of bilateral background hyperplasia and this could predict less favorable post-operative outcome with residual hypertension (35, 87).

## Increased but Variable Expression of MC2R in PA

The explanation for the increased role of ACTH in the regulation of aldosterone in PA may be secondary to the overexpression and function of MC2R in this condition. The expression of MC2R mRNA was shown to be upregulated in human adrenocortical neoplasms specifically in functional adenomas in contrast to non-functioning adenomas and carcinomas (89). More specifically, a few studies have demonstrated increased eutopic expression of MC2R assessed by RT-PCR or transcriptome studies in resected aldosteronomas as compared to cortisol-secreting adenomas, non-functional adenomas, and adrenocortical carcinomas (90–92). A particularly pertinent informative study included 15 adrenal tumors (14 APA and 1 BAH); MC2R mRNA levels were increased by a mean of 3.9-fold in those tissues compared to normal adrenal (18). However great variability existed in the level of expression in each tumor as 4 had lower levels than normal (0.3-fold to 0.7-fold), while those with increased expression varied between 1.4- and 20.6-fold compared to normal. The data are limited to mRNA expression without available measurements at the protein levels (no good specific MC2R antibody), but correlated well with the *in vivo* increased response to ACTH administration. There is almost no data on MC2R expression in BAH as those patients are usually not operated, but in the only case with BAH studied by this group MC2R was 20-fold increased. These data appear to be compatible with the findings that the majority of patients stimulated with ACTH during AVS will have concordant results before and after ACTH as the majority overexpress MC2R; however, the 28% of discordant results we found (87) may be explained by cases where MC2R are relatively decreased in the dominant adenoma but is present in adjacent hyperplasia. This hypothesis remains to be validated in prospective studies.

## Other Hormones and Aberrant Receptors Regulating Aldosterone Secretion in Primary Aldosteronism

Adrenocorticotropin hormone is not the exclusive trigger of aldosterone secretion since several other hormones have a role in the pathophysiology of PA in addition to the ion channels mutations. Serotonin plays a significant role in aldosterone synthesis in normal physiological and in PA. The administration of serotonin 5-HT_4_ agonists such as metoclopramide, cisapride, and tegaserod resulted in higher stimulation of aldosterone in PA as compared to the physiological moderate increase in normal individuals (18, 93, 94). Whereas non-specific inhibitors of 5-HT such as cyproheptadine and ketanserin produced only minor and transient inhibition of aldosterone secretion in aldosteronomas (95, 96), specific 5-HT_4_R antagonists such as GR113808 were potent inhibitors of basal- and cisapride-induced aldosterone secretion (93). Chromaffin cells, endothelial cells, nerve terminals, and cells of the immune system are localized in the immediate vicinity of ZG cells and can secrete various factors to control aldosterone secretion (97). Local release of 5-HT by perivascular mast cells (MC) can activate 5-HT_4_R expressed in ZG cells and consequently stimulate aldosterone production (98). A role of MC in tumorigenesis was proposed (99, 100). The density of MC was found to be increased in APA tissues compared with normal adrenals (101). As the 5-HT_4_R have been found to be overexpressed in the majority of APA (but variable as MC2R) (17, 18, 102) and the ligand may be locally overexpressed also, a paracrine loop of regulation of aldosterone production appears to be present.

The compelling evidence supporting that various aberrant GPCR are frequently expressed in bilateral macronodular adrenal hyperplasia and Cushing’s syndrome (103) led many researchers to investigate the presence of aberrant GPCR in PA. Adrenal production of aldosterone in APA and BAH was found to be under the influence of aberrant GPCR and their ligands, as demonstrated by *in vivo* and *in vitro* studies (104, 105). The expression of ectopic receptors, which are usually not expressed at significant levels in normal ZG cells include those for glucose-dependent insulinotropic peptide (GIPR) (106), luteinizing hormone/human chorionic gonadotropin (LH–hCG R) (18, 106–112), β-adrenergic receptors (β-AR) (113), vasopressin (V1-AVPR) (18, 106, 114, 115), glucagon (glucagon receptor), TRH (TRH R) (18, 112, 116) and Endothelin-1 ET_A_ and ET_B_ receptors (117). Using a microarray approach in 10 aldosteronomas compared with five normal adrenals and 13 cortisol-secreting adenomas, the six GCPRs with highest increase in expression included LHCGR, 5-HT_4_R, GnRHR, glutamate receptor metabotropic 3, endothelin receptor ET_B_ receptors, and MC2R (92). Table 1 summarizes the different types of aberrant eutopic or ectopic GPCR involved in aldosterone excess in PA. Co-expression of multiple aberrant GPCR was also reported; renin-independent stimulation of aldosterone secretion was observed *in vivo* following mixed meal, oral glucose, or administration of GIP, vasopressin, and tegaserod in a patient with unilateral source of PA (106). On the other hand, co-secretion of aldosterone and cortisol due to aberrant expression of GPCR was noted; in a patient with BMAH and β-AR-aberrant expression, isoproterenol stimulated both cortisol and aldosterone production (113).

**Table 1:** **Types of GPCR involved in aldosterone hypersecretion in patients with PA**.

Aberrant receptor	Phenotype	*In vivo* screening protocol	Targeted medical therapy
MC2R (eutopic) (90–92)	ACTH-dependent hyperaldosteronism	Cosyntropin	
GIP receptor (ectopic) (102)	Food-dependent hyperaldosteronism	Mixed meal	Octreotide, pasireotide
		Oral glucose	
			GIPR antagonist
Vasopressin receptor (ectopic) (18, 102, 114, 115)	Upright posture-dependent hyperaldosteronism	Upright posture AVP/desmopressin	Specific AVP receptors antagonist
β-adrenergic receptor (ectopic) (113)	Upright posture	Upright posture	β-blockers
	Insulin-induced hypoglycemia Exercise/stress test hyperaldosteronism	Isoproterenol (β1-agonist)	
GnRH receptor, LH/hCG receptor (ectopic) (18, 102, 107–112)	Luteal phase of ovarian cycle/Pregnancy (transient)	GnRH, hCG, Recombinant LH	Long-acting GnRH agonist (leuprolide acetate*)*
	Postmenopausal (persistent)-dependent hyperaldosteronism		
5-HT_4_ receptor (eutopic) (18, 93, 94, 102)	Serotonin-dependent dependent	5-HT_4_ receptor agonists (metoclopramide, cisapride, tegaserod)	5-HT_4_ receptor antagonist (GR113808)
Glucagon receptor (ectopic) (18)	Hypoglycemia?	Intravenous glucagon	Octreotide
TRH receptor (ectopic) (18, 112, 116)	Hypothyroidism		
Endothelin-1 A and B receptors (ectopic) (117)			

Activating somatic *CTNNB1* (β-catenin) mutations have now been identified in tumors of three women with APAs, two who presented during pregnancy and one after menopause (118). All three had heterozygous activating mutations of *CTNNB1* and expressed aberrant LHCG and GNRH receptors at levels 100-fold higher than in other APAs. It was shown that the *CTNNB1* mutation led to activation of the WNT pathway; it was suggested that could be the cause of dedifferentiation of gonadal progenitor cells present in the adrenal tissues with increased expression of gonadal receptors. It is thought that the high levels of endogenous human chorionic gonadotropin (hCG) during pregnancy and of gonadotropin-releasing hormone (GnRH) and luteinizing hormone (LH) after menopause led to the identification of APAs in these patients (118).

It is currently unclear whether these aberrant regulatory secretory mechanisms by ACTH and other hormones and the overexpression of their GPCR in PA are secondary to unknown proliferative mechanisms or are primary and at least partially responsible for the abnormal proliferation, the initiation of diffuse BAH. However, they clearly play a role in aldosterone secretion which is not autonomous.

## Conclusion

Our understanding of the increased occurrence and complexity of molecular etiology and unique signature in each case of PA has progressed greatly in recent years. The increased role of ACTH, of the variable expression of MC2R, and of other aberrant GPCR in PA should receive further attention in the future. The development of effective antagonists to MC2R and other aberrant GPCR could eventually offer interesting alternatives in patients with bilateral sources of excess aldosterone in combination with better antagonists of the mineralocorticoid receptor.

## Author Contributions

NG, IB, and AL contributed to the conception and design of the manuscript as well as to drafting the review article and they all provided final approval of the version to be published.

## Conflict of Interest Statement

The authors declare that the research was conducted in the absence of any commercial or financial relationships that could be construed as a potential conflict of interest.

## Abbreviations

β-AR, β-adrenergic receptors; 5-HT_4_R, serotonin receptor; AC, adenylate cyclase; ACTH, adrenocorticotropin hormone; APA, aldosterone-producing adenoma; APA, aldosterone-producing adenoma; APCCs, aldosterone-producing cell clusters; AT-1 receptor, angiotensin II type 1 receptor; ATP, adenosine triphosphate; AVS, adrenal venous sampling; BAH, bilateral adrenal hyperplasia; cAMP, cyclic adenosine monophosphate; CREB, cAMP response element binding protein; CYP11B1, cytochrome P450 family 11 subfamily B member 1 encodes 11-beta hydroxylase; CYP11B2, cytochrome P450 family 11 subfamily B member 2 encodes aldosterone synthetase; ET, endothelin-1; FH, familial hyperaldosteronism; FST, fludrocortisone suppression test; GIP, glucose-dependent insulinotropic peptide; GPCR, G-protein-coupled receptor; GRA, glucocorticoid-remediable aldosteronism; Gs-α, G stimulatory α subunit; LH–hCG, luteinizing hormone/human chorionic gonadotropin; MC2R, melanocortin type 2 receptor; mRNA, messenger ribonucleic acid; PA, primary aldosteronsim; PAC, plasma aldosterone; PKA, protein kinase; RAS, rennin–angiotensin system; RT-PCR, reverese transcriptase-polymerase chain reaction; StAR, steroidogenic acute regulatory protein; TRH, thyrotropin releasing hormone; UA, unilateral adrenalectomy; V1-AVPR, vasopressin receptor type 1; ZF, zona fasciculata; ZG, zona glomerulosa.
